# Effect of Individual Omega-3 Fatty Acids on the Risk of Prostate Cancer: A Systematic Review and Dose-Response Meta-Analysis of Prospective Cohort Studies

**DOI:** 10.2188/jea.JE20140120

**Published:** 2015-04-05

**Authors:** Yuan-Qing Fu, Ju-Sheng Zheng, Bo Yang, Duo Li

**Affiliations:** Department of Food Science and Nutrition, Zhejiang University, Hangzhou, China

**Keywords:** prostate cancer, omega-3 fatty acid, meta-analysis, prospective cohort

## Abstract

Epidemiological studies have suggested inconsistent associations between omega-3 polyunsaturated fatty acids (n-3 PUFAs) and prostate cancer (PCa) risk. We performed a dose-response meta-analysis of prospective observational studies investigating both dietary intake and circulating n-3 PUFAs and PCa risk. PubMed and EMBASE prior to February 2014 were searched, and 16 publications were eligible. Blood concentration of docosahexaenoic acid, but not alpha-linolenic acid or eicosapentaenoic acid, showed marginal positive association with PCa risk (relative risk for 1% increase in blood docosahexaenoic acid concentration: 1.02; 95% confidence interval, 1.00–1.05; I^2^ = 26%; *P* = 0.05 for linear trend), while dietary docosahexaenoic acid intake showed a non-linear positive association with PCa risk (*P* < 0.01). Dietary alpha-linolenic acid was inversely associated with PCa risk (relative risk for 0.5 g/day increase in alpha-linolenic acid intake: 0.99; 95% confidence interval, 0.98–1.00; I^2^ = 0%; *P* = 0.04 for linear trend), which was dominated by a single study. Subgroup analyses indicated that blood eicosapentaenoic acid concentration and blood docosahexaenoic acid concentration were positively associated with aggressive PCa risk and nonaggressive PCa risk, respectively. Among studies with nested case-control study designs, a 0.2% increase in blood docosapentaenoic acid concentration was associated with a 3% reduced risk of PCa (relative risk 0.97; 95% confidence interval, 0.94–1.00; I^2^ = 44%; *P* = 0.05 for linear trend). In conclusion, different individual n-3 PUFA exposures may exhibit different or even opposite associations with PCa risk, and more prospective studies, especially those examining dietary n-3 PUFAs and PCa risk stratified by severity of cancer, are needed to confirm the results.

## INTRODUCTION

Prostate cancer (PCa) is the second most common cancer and the sixth leading cause of cancer-related death among men around the world, accounting for 14% of the total new cancer cases and 6% of the total cancer deaths in men in 2008.^1^ Over the past decades, a large number of epidemiological studies have suggested that a healthy diet or lifestyle played an important role in the prevention of cancer, as genetic factors were attributed to less than 10% of PCa.^2^^,^^3^ Dietary fatty acids, especially n-3 polyunsaturated fatty acids (n-3 PUFAs), are one of the most intensively studied dietary factors closely related with PCa risk. n-3 PUFAs mainly include alpha-linolenic acid (ALA, 18:3n-3), eicosapentaenoic acid (EPA, 20:5n-3), docosapentaenoic acid (DPA, 22:5n-3), and docosahexaenoic acid (DHA, 22:6n-3). ALA from plant food sources is partly converted to EPA and DPA in the body, whereas EPA, DPA, and DHA are categorized as marine n-3 PUFAs because they are largely from marine-based animal food sources (ie, fatty fish).^4^ Besides dietary exposure of n-3 PUFAs, blood concentration of n-3 PUFAs should also be examined for its association with PCa, because blood biomarkers of n-3 PUFAs are objective, and the reliability does not depend on the accuracy of memories, awareness of fat intake, or willingness to report details of one’s diet.

A considerable number of studies, including both animal and in vitro cell studies, have demonstrated that n-3 PUFAs are the most promising type of nutrients to inhibit or curtail carcinogenesis and reduce PCa risk.^5^^–^^7^ Results from observational studies, however, have been inconsistent. Several case-control and prospective cohort studies have suggested a positive association of both circulating ALA and dietary ALA with PCa risk.^8^^–^^11^ On the contrary, there are also multiple case-control or prospective cohort studies reporting negative associations or no association between ALA (circulating and dietary) and PCa risk.^12^^–^^14^ Moreover, accumulating prospective studies have suggested that long chain n-3 PUFAs showed inverse, null, or even positive association with PCa risk.^15^^–^^18^ Even systematic reviews and meta-analyses summarizing the relationship of n-3 PUFAs with PCa risk have yielded opposite conclusions, though none of them were dose-response meta-analyses of prospective studies quantifying the association between n-3 PUFAs (dietary or circulating) with PCa risk.^19^^–^^22^

Since the most recently published systematic review, at least four new studies with large sample sizes have been published and made available. Therefore, we performed a dose-response meta-analysis to estimate the trend and quantify the association for both dietary intakes and blood concentration of individual n-3 PUFAs with PCa risk based on prospective studies only.

## MATERIALS AND METHODS

### Search strategy and selection criteria

The standard MOOSE criteria^23^ for conducting and reporting meta-analysis of observational studies were followed in the present study. We searched two databases (PubMed and EMBASE) up to February 2014. The search strategy was based on the following title/abstract key words: (‘fat’ OR ‘fatty acid’ OR ‘docosahexaenoic acid’ OR ‘eicosapentaenoic acid’ OR ‘docosapentaenoic acid’ OR ‘alpha-linolenic acid’ OR ‘polyunsaturated fatty acid’ OR ‘omega-3 fatty acid’ OR ‘n-3 fatty acid’) AND (‘prostate cancer’ OR ‘prostate neoplasms’) (eTable 1). We also reviewed the reference lists of relevant studies to identify studies that might have been missed. We contacted authors for detailed information of primary studies when needed.

Literature search and data extraction were independently conducted by two investigators (YQ-F and JS-Z), and discrepancies were resolved by discussion with a third investigator (DL). Inclusion criteria included prospective study design (including prospective cohort, nested case-control, and case-cohort studies); exposure of interest (dietary n-3 PUFAs or blood n-3 PUFAs concentrations); endpoint (incident PCa in males); and reporting of risk estimate (relative risk, odd ratio, or hazard ratio) of PCa with corresponding 95% confidence intervals (CIs) for individual n-3 PUFA exposure. Retrospective or cross-sectional studies, animal or cell culture studies, reviews, editorials, and commentaries were all excluded from the present study.

### Data extraction

Detailed data relating to participants (study population and region, age of participants, number of cases and non-cases), characteristics of study (first author’s name, study design, follow-up period, method of n-3 PUFA measurement, adjusted confounding factors), risk estimates with the most adjustment and corresponding 95% CIs for each category of n-3 PUFA exposure were extracted. Relative risk (RR) was used for risk estimates, and odd ratio and hazard ratio were treated as RR directly as reported elsewhere.^24^

We assessed study quality using the Newcastle-Ottawa criteria.^25^ The criterion contains selection domain (4 points), comparability domain (2 points), and outcomes (for cohort studies) or exposures (for case-control studies) domain (3 points). Scores of 0–3, 4–6, and 7–9 were regarded as low, moderate, and high quality, respectively.

### Data synthesis

For studies evaluating the association between dietary n-3 PUFAs and PCa risk, dose-response analysis was only conducted among studies with exposure units reported as or transformable to g/day; we did not conduct dose-response analysis for studies reporting units as %energy/day due to the limited number of studies. One study,^17^ reporting the unit for fatty acid intake category as grams/1000 kcal, was transformed to g/day assuming an average energy intake of 2000 kcal/day in this population.^12^ Any results stratified by stage or grade of PCa were treated as separate reports and combined first before being pooled into the overall meta-analysis. We did not carry out dose-response analysis for dietary individual n-3 PUFA and PCa subgroups (ie, aggressive or non-aggressive), because very few studies (not more than three) reported the findings stratified by stage of cancer. We performed a 2-stage random-effects dose-response meta-analysis first to examine a potential curvilinear association between dietary n-3 PUFA exposure and risk of PCa, using restricted cubic splines with three knots at fixed percentiles (25%, 50%, and 75%) of dietary n-3 PUFA distribution.^26^ The median or mean value of n-3 PUFA exposure in each category was used as the corresponding dose of exposure. If the median or mean value was not available, the midpoint of the upper and lower boundary was considered the dose of each category. If the highest category was open-ended, we assumed the category to be of the same width as the closest category. For only one study, the median value (or average or range) of the fatty acids in each category was not reported in the publication, we obtained the data by contacting the authors.^27^ A *P*-value for non-linearity was calculated by testing the null hypothesis that the coefficient of the second spline was equal to zero. If the non-linear association between a specific individual n-3 PUFA and PCa risk was found to be non-significant, the trend estimation from the correlated estimates for log RR across categories of individual dietary n-3 PUFA exposure, assuming a linear relationship, was performed, and the RR with 95% CI for a specific increment of individual n-3 PUFA intake was computed using generalized least squares regression, as described by Greenland and Longnecker^28^ and Orsini and colleagues.^29^

For studies examining the association between blood n-3 PUFA concentration and PCa risk, all reported the concentration of blood n-3 PUFA as ‘% total fatty acid’, and some of the studies also examined the findings stratified by grade or stage of cancer. We defined low-grade (Gleason scores ≤7) and localized tumors as nonaggressive, while treating high-grade (Gleason scores >7) and advanced tumor cases as aggressive, according to the classification described by Orsini.^26^ The dose-response analysis was separately conducted for aggressive, non-aggressive, and total PCa risk. For one study, the reported PCa risks were stratified by grade or stage of cancer and we obtained the RRs for total PCa by contacting the authors.^14^ As described previously, we first performed a 2-stage random-effects dose-response meta-analysis to examine a potential non-linear association between any individual blood n-3 PUFA concentration and PCa risk, using restricted cubic splines with three knots at fixed percentiles (25%, 50%, and 75%) of n-3 PUFA distribution.^26^ If significant non-linear association between a specific individual n-3 PUFA and PCa was not found, the trend estimation, assuming a linear relationship, was performed, and the RR with 95% CI for a specific increment of individual n-3 PUFA concentration was computed using generalized least squares regression, as described above. For only one study without sufficient information to estimate the distribution, we used variance-weighted least squares regression for the dose-response estimation.^30^

All dose-response analyses were separately conducted for ALA, EPA, DHA, and DPA. Study heterogeneity was assessed using the I^2^ statistic. An I^2^ statistic <25% indicated low heterogeneity, 25%–75% indicated moderate heterogeneity, and >75% indicated high heterogeneity. Subgroup analyses were conducted to examine sources of study heterogeneity and the influence of potential residual confounding factors, such as age, BMI, alcohol consumption, and education. If the number of reports in each subgroup was very limited, we did not estimate dose-response trends for these subgroups. Sensitivity analysis was conducted by omitting one study at a time and examining the influence of each individual study on the pooled RR. Publication bias was evaluated by visual inspection of a funnel plot and use of Begg’s regression test (significant at *P* < 0.05). Stata version 12 (StataCorp LP, College Station, TX, USA) was used for all statistical analyses.

## RESULTS

### Literature search

**Figure 1.**
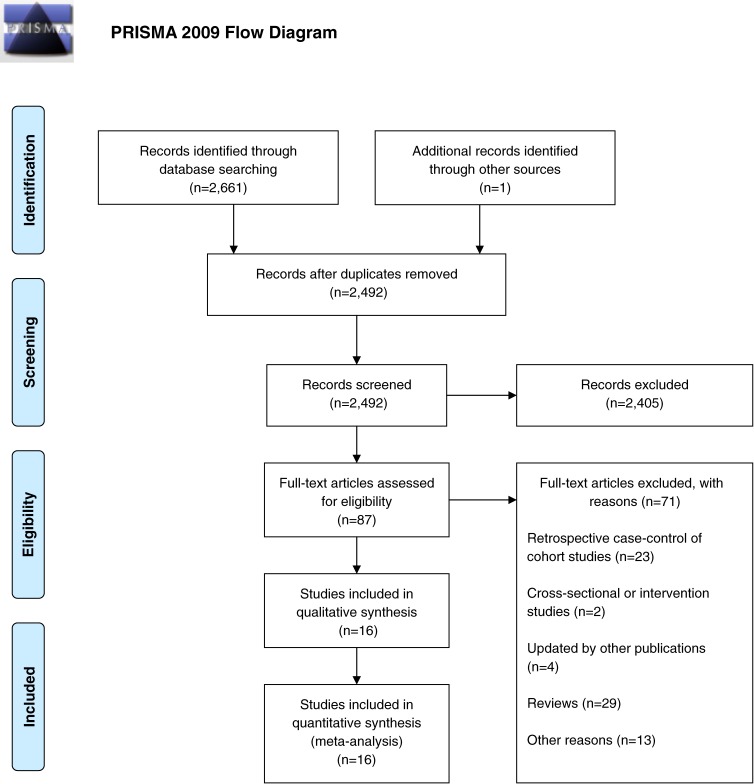
Flow diagram for selection of studies in the meta-analysis.

Through full-text examination of 87 potential publications, we identified 16 eligible publications, including 35 828 PCa cases and 840 242 participants, from 14 independent prospective cohort studies (Figure 1). Among these articles, 10 were from the USA,^13^^–^^15^^,^^17^^,^^31^^–^^36^ 5 were from Europe,^8^^,^^16^^,^^18^^,^^30^^,^^37^ and 1 was from Australia.^27^ Furthermore, 14 articles reported the association of marine n-3 PUFA exposure with PCa risk, and 14 articles described the association of alpha-linolenic acid with PCa risk.

### Study characteristics

The 16 eligible articles reported on prospective studies with mean follow-up duration ranging from 1.9 to 16 years. Among these studies, 7 were nested case-control studies, 6 were prospective cohort studies, and 3 were case-cohort studies. For one cohort, RR for n-3 PUFA intakes and circulating n-3 PUFA concentration were reported in two different publications.^17^^,^^36^ For one cohort, RR for ALA intake with PCa risk was reported twice in two different publications, and the data from the report with longer follow-up were used,^32^ while RR for marine n-3 fatty acids was only reported in the other article.^33^ The general characteristics of all included studies are listed in eTable 2 and eTable 3, the adjusted covariates for included studies are listed in eTable 4, and the case ascertainment methods of included studies are listed in eTable 5. Quality assessment indicated that the average score of included studies was 7.3, and the scores were 6 or above (moderate or high quality) for all studies (eTable 6).

### Association between ALA and PCa risk

Fourteen articles^8^^,^^13^^–^^15^^,^^17^^,^^18^^,^^27^^,^^30^^–^^32^^,^^34^^–^^37^ from 13 independent cohorts, involving 35 186 PCa events and 781 963 participants, were included for the analysis of association between ALA exposure and PCa risk.

**Figure 2.**
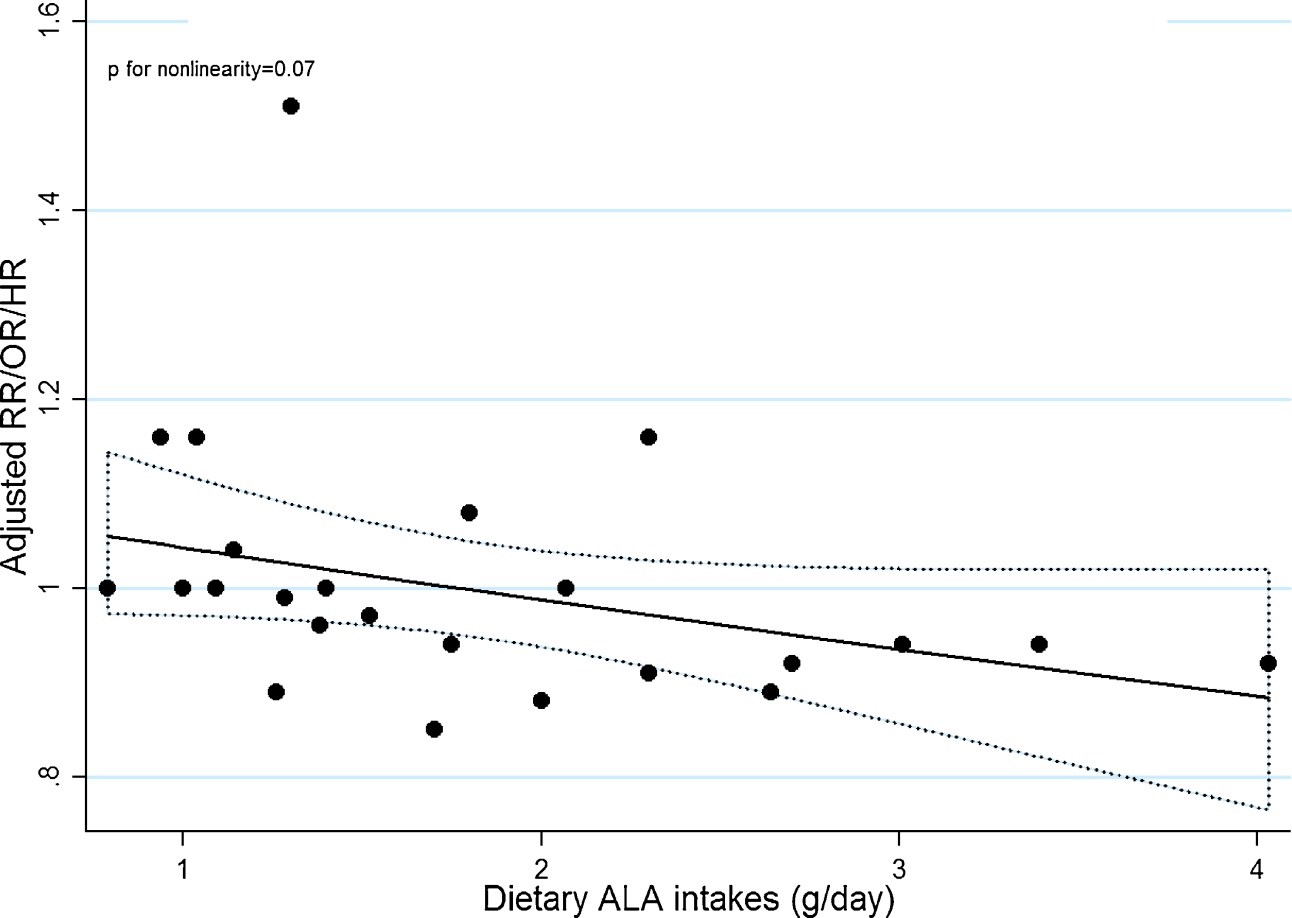
Dose-response relationship between dietary ALA intake and PCa risk. The dotted line represents the 95% confidence limits for the fitted curve.

**Figure 3.**
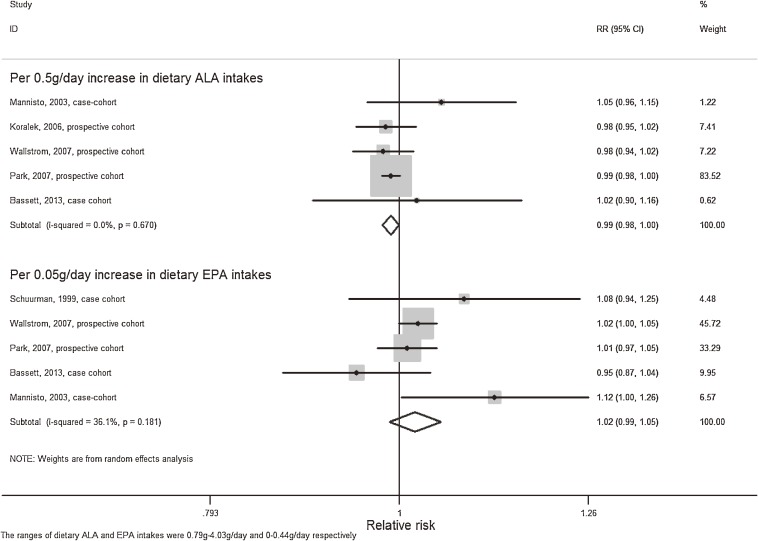
Meta-analysis of PCa risk per 0.5 g/day increment of dietary ALA and per 0.05 g/day increment of dietary EPA. A random-effects model was used to estimate overall relative risk. Grey squares stand for study-specific relative risks, with the square size reflecting the corresponding weight and horizontal bars reflecting 95% confidence intervals.

Five articles^13^^,^^17^^,^^18^^,^^27^^,^^30^ that reported intakes of ALA as g/day or grams/1000 kcal/day were eligible for the dose-response analysis. Using a restricted cubic splines model, we did not find a significant curvilinear association (*P* = 0.07) between dietary ALA intake and PCa risk (Figure 2). Begg’s test indicated no significant publication bias (*P* = 0.81). The summary RR of PCa for an increase of 0.5 g/day was 0.99 (95% CI, 0.98–1.00), and no heterogeneity was observed (I^2^ = 0%); however, this result should be interpreted with caution because one study dominated the results (Figure 3).^17^ We did not perform dose-response analysis for those articles examining risk of PCa stratified by grade or stage because only three articles reporting risk of aggressive PCa and one article reporting risk of non-aggressive PCa were eligible.

**Figure 4.**
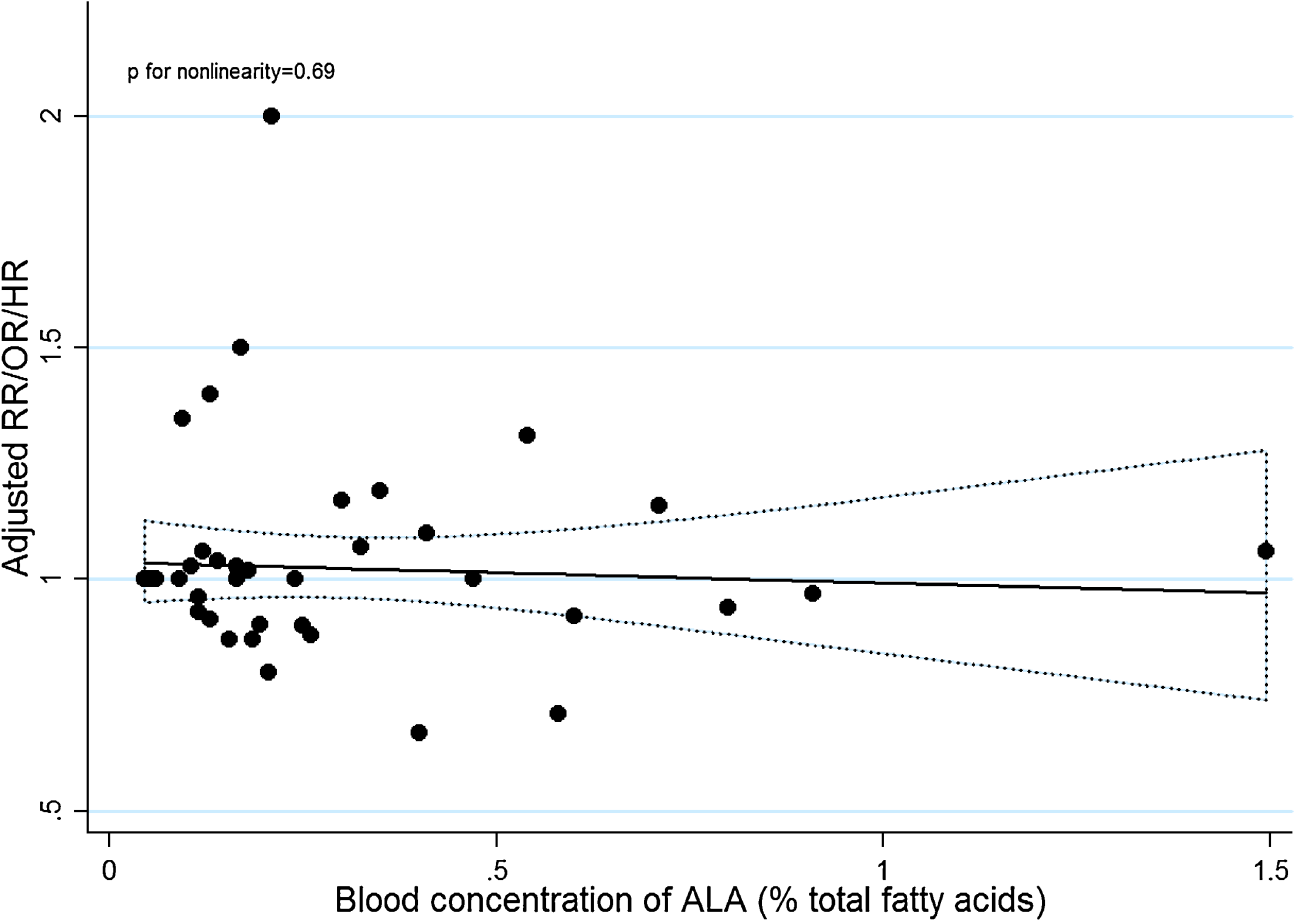
Dose-response relationship between blood ALA concentration and PCa risk. The dotted line represents the 95% confidence limits for the fitted curve.

**Figure 5.**
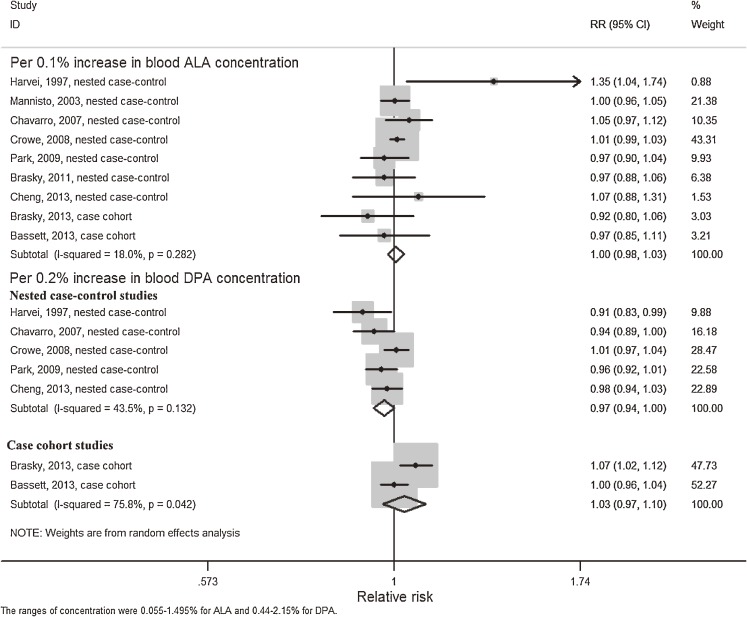
Meta-analysis of PCa risk per 0.1% increase in blood ALA concentration and per 0.2% increase in blood DPA concentration. A random-effects model was used to estimate overall relative risk. Grey squares stand for study-specific relative risks, with the square size reflecting the corresponding weight and horizontal bars reflecting 95% confidence intervals.

Nine articles^14^^,^^15^^,^^27^^,^^30^^,^^34^^–^^37^ were eligible for the trend estimation between blood ALA and risk of PCa and no potential curvilinear association (*P* = 0.69) was examined (Figure 4). Dose-response analysis did not find an association with PCa risk per 0.1% increment of blood ALA concentration (RR 1.00; 95% CI, 0.98–1.03; I^2^ = 18%) (Figure 5) and funnel plot visualization and the Begg’s test indicated no publication bias (*P* = 0.80 for Begg’s test). For PCa stratified by grade or stage of cancer, we did not observe a significant linear association (RR 0.98 per 0.1% increase in blood ALA concentration; 95% CI, 0.87–1.11; I^2^ = 42%) between blood ALA concentration and aggressive PCa from 6 eligible studies. For non-aggressive PCa, of which only 4 studies were included, we found a statistically significant non-linear association with blood ALA (*P* < 0.01), indicating a protective role of ALA (eTable 7).

### Association between marine n-3 PUFA and PCa risk

Fourteen articles^8^^,^^14^^–^^18^^,^^27^^,^^30^^,^^31^^,^^33^^–^^37^ from 13 independent cohort studies, involving 33 351 PCa events and 806 987 participants, reported an association between marine n-3 PUFA and PCa risk. Among the included studies, 14 articles reported RR for EPA, 14 articles reported RR for DHA, and 7 articles reported RR for DPA. We performed dose-response analyses for EPA, DHA, and DPA separately from identified studies.

**Figure 6.**
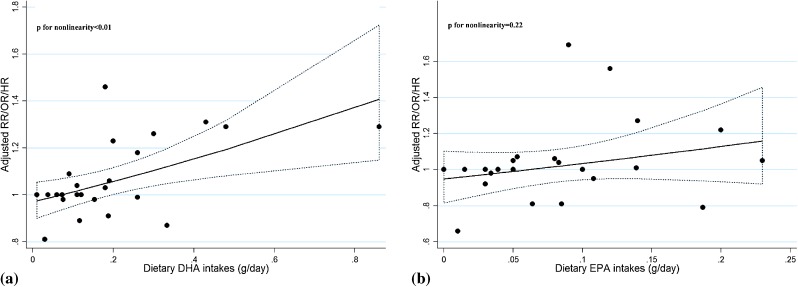
Dose-response relationship for dietary DHA and EPA intakes with PCa risk. The dotted line represents the 95% confidence limits for the fitted curve.

Five studies^16^^–^^18^^,^^27^^,^^30^ that reported both dietary DHA and EPA as g/day or g/energy/day were eligible for the dose-response analysis, and no significant publication bias was observed (*P* = 0.58 and *P* = 0.81 for Begg’s test respectively). A significant curvilinear association was observed (*P* < 0.01) between dietary DHA exposure and PCa risk, which indicated that increased DHA intake was associated with higher risk of PCa (Figure 6a). Therefore, trend estimation, assuming a linear relationship, was not conducted. For dietary EPA exposure, there was no curvilinear association with PCa risk (*P* = 0.22) (Figure 6b), and trend estimation did not find any significant relationship with PCa risk per 0.05 g/day increment of EPA intake (RR 1.02; 95% CI, 0.99–1.05, I^2^ = 36.1%) (Figure 3). We did not examine the dose-response association between dietary DHA or EPA exposure and PCa risk stratified by grade or stage of cancer because only three articles reporting risk of aggressive PCa and one article reporting risk of non-aggressive PCa were eligible.

**Figure 7.**
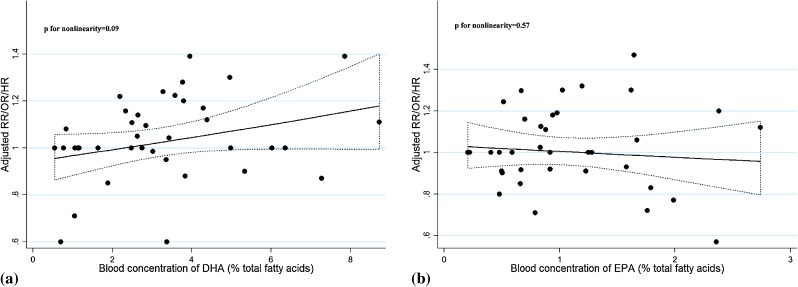
Dose-response relationship for blood concentration of DHA and EPA with PCa risk. The dotted line represents the 95% confidence limits for the fitted curve.

**Figure 8.**
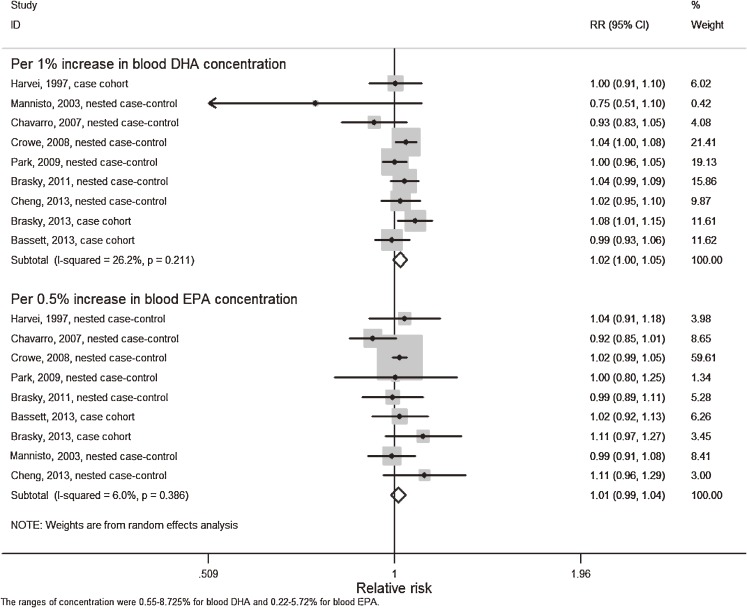
Meta-analysis of PCa risk per 1% increase in blood DHA concentration and per 0.5% increase in blood EPA concentration. A random-effects model was used to estimate overall relative risk. Grey squares stand for study-specific relative risks, with the square size reflecting the corresponding weight and horizontal bars reflecting 95% confidence intervals.

Nine studies^14^^,^^15^^,^^27^^,^^30^^,^^34^^–^^37^ that reported both blood level of DHA and EPA exposure were included for the dose-response analysis. There was no significant curvilinear association between blood concentration of DHA or EPA and PCa risk (*P* = 0.09 and *P* = 0.57, respectively) (Figure 7a and Figure 7b) and no significant publication bias indicated by Begg’s test (*P* = 0.176 for DHA and *P* = 0.175 for EPA, respectively). The trend estimation analysis indicated a borderline significant positive association with PCa risk per 1% increase of blood DHA concentration (*P* = 0.11) but not per 0.5% increase of EPA concentration (*P* = 0.32) (Figure 8). However, when stratified by grade or stage, significant non-linear relationships were found for blood DHA concentration with non-aggressive PCa risk (*P* < 0.01) and for blood EPA concentration with aggressive PCa risk (*P* < 0.01), both indicating increased PCa risk with higher concentration of DHA or EPA (Table 1 and Table 2).

**Table 1.** Subgroup analyses per 1% increment^a^ of blood DHA^b^ concentration and risk of PCa

Subgroup factor	Number of studies	Relative risk (95% CI)	Heterogeneity within sub-group		Heterogeneity between sub-groups	Nonlinearity
		
			I^2^	Degree	*P* value	*P* value
Overall analysis	9	1.02 (1.00, 1.05)	26.2%		—	0.11
Study design						
Nested case-control	7	1.02 (0.99, 1.05)	17%	Low	0.61	0.09
Case-cohort	2	—	—		—	—
Grade or stage of cancer						
Aggressive	5	1.08 (0.99, 1.18)	84%	High	—	0.83
Non-aggressive	4		—	—	—	<0.01
Follow-up duration						
≤7.4 years	6	1.03 (1.00, 1.06)	25%	Low	0.08	0.88
>7.4 years	3	—	—	—		0.04
Regions						
USA	5	1.02 (0.99, 1.06)	38%	Moderate	0.64	0.99
European countries	3	—	—	—		0.02
Australia	1	—	—	—	—	—
Covariate adjustment						
Adjusted for age	5	1.02 (0.99, 1.06)	38%	Moderate	0.98	0.99
Not adjusted for age	4	1.01 (0.97, 1.06)	33%	Moderate		0.36
Adjusted for BMI	4	1.03 (1.00, 1.05)	0%	Low	0.61	0.41
Not adjusted for BMI	5	1.00 (0.94, 1.07)	53%	Moderate		0.31
Adjusted for alcohol consumption	4	1.03 (1.00, 1.06)	0%	Low	0.43	0.06
Not adjusted for alcohol consumption	5	1.01 (0.95, 1.06)	52%	Moderate		0.38
Adjusted for smoking status	4	1.01 (0.98, 1.05)	31%	Moderate	0.85	0.20
Not adjusted for smoking status	5	1.01 (0.99, 1.03)	0%	Low		0.26
Adjusted for family history of PCa	4	0.99 (0.93, 1.07)	50%	Moderate	0.92	0.05
Not adjusted for family history of PCa	5	1.02 (1.00, 1.05)	17%	Low		0.67
Risk expression						
Hazard/rate ratio	2	—	—		—	—
Relative risk	2	—	—		—	—
Odds ratio	5	1.01 (0.99, 1.04)	0%	Low	0.41	0.30

BMI, body mass index; CI, confidence interval; PCa, prostate cancer.

^a^The range of blood DHA concentration in the included studies is 0.55% to 8.73%.

^b^DHA, docosahexaenoic acid.

**Table 2.** Subgroup analyses per 0.5% increment^a^ of blood EPA^b^ concentration and risk of PCa

Subgroup factor	Number of studies	Relative risk (95% CI)	Heterogeneity within subgroup		Heterogeneity between subgroups	Nonlinearity
		
			I^2^	Degree	*P* value	*P* value
Overall analysis	9	1.01 (0.99, 1.04)	6%	Low	—	0.88
Study design						
Nested case-control	7	1.01 (0.98, 1.04)	12%	Low	0.39	0.50
Case-cohort	2	—	—		—	—
Grade or stage of cancer						
Aggressive	5	—	—	—	—	<0.01
Non-aggressive	4	1.02 (0.98, 1.07)	11%	Low		0.23
Follow-up duration						
≤7.4 years	6	1.02 (1.00, 1.04)	0%	Low	0.19	0.40
>7.4 years	3	0.98 (0.91, 1.06)	37%	Moderate		0.39
Regions						
USA	5	1.01 (0.94, 1.10)	47%	Moderate	0.49	<0.01
European countries	3	1.02 (0.99, 1.04)	0%	Low		0.56
Australia	1	—	—	—	—	—
Covariate adjustment						
Adjusted for age	5	1.01 (0.94, 1.10)	46%	Moderate	0.49	0.01
Not adjusted for age	4	1.02 (0.99, 1.04)	0%	Low		0.16
Adjusted for BMI	4	1.02 (1.00, 1.05)	0%	Low	0.34	0.20
Not adjusted for BMI	5	1.00 (0.95, 1.06)	33%	Moderate		0.38
Adjusted for alcohol consumption	4	1.02 (1.00, 1.05)	0%	Low	0.27	0.19
Not adjusted for alcohol consumption	5	1.00 (0.94, 1.06)	30%	Moderate		0.44
Adjusted for smoking status	4	1.01 (0.95, 1.07)	51%	Moderate	0.93	0.08
Not adjusted for smoking status	5	1.02 (0.96, 1.07)	0%	Low		0.20
Adjusted for family history of PCa	4	1.00 (0.95, 1.04)	41%	Moderate		0.42
Not adjusted for family history of PCa	5	1.04 (0.98, 1.10)	0%	Low		0.53
Risk expression						
Hazard/rate ratio	2	—	—		—	—
Relative risk	2	—	—		—	—
Odds ratio	5	1.02 (0.96, 1.07)	0%	Low	0.99	0.40

BMI, body mass index; CI, confidence interval; PCa, prostate cancer.

^a^The range of blood EPA concentration in the included studies is 0.22% to 5.72%.

^b^EPA, eicosapentaenoic acid.

**Figure 9.**
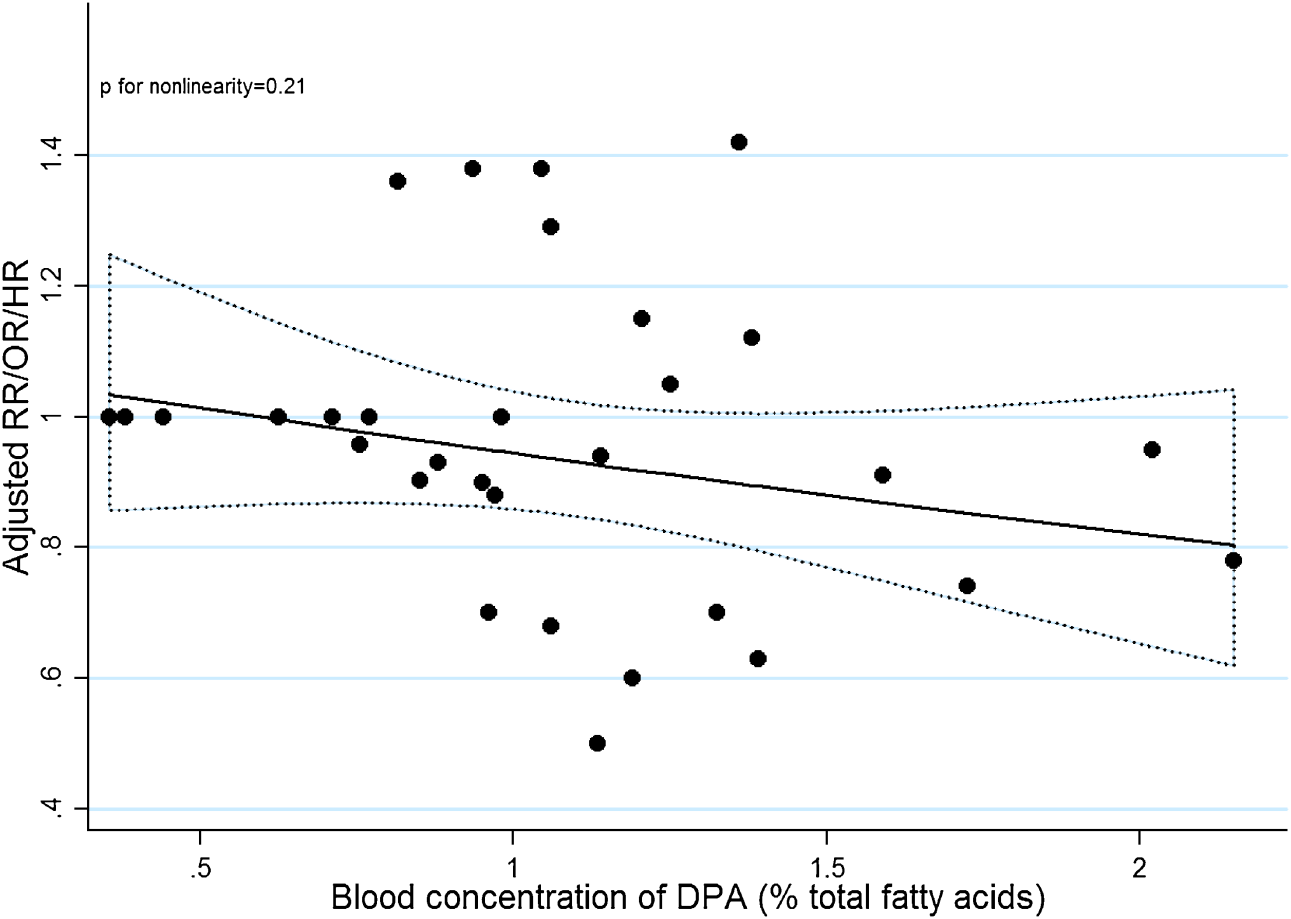
Dose-response relationship for blood DPA concentration with total PCa risk. The dotted line represents the 95% confidence limits for the fitted curve.

No significant curvilinear association between blood concentration of DPA and PCa risk was observed (*P* = 0.21) (Figure 9). After excluding two studies with case cohort design, the trend estimation analysis indicated a statistically significant negative association for a 0.2% increment of blood DPA concentration with PCa risk (RR 0.97; 95% CI, 0.94–1.00; I^2^ = 43.5%) (Figure 4).

### Sensitivity and subgroup analysis

For dietary ALA exposure, sensitive analysis indicated that excluding the only study^30^ for which trend estimation was carried out using variance-weighted least squares regression didn’t change the pooled RR estimate (RR 0.99; 95% CI, 0.98–1.00; I^2^ = 0%) per 0.5 g/day increments. However, exclusion of the study^17^ dominating the overall outcomes did make the pooled RR estimate non-significant. Therefore, the significant inverse association between ALA intake and PCa risk should be interpreted with caution. Sensitivity analysis also indicated that omitting any individual study did not substantially change the significance of the non-linear association with PCa risk for dietary DHA intakes and did not change the pooled RR estimates for dietary EPA intakes. However, much lower heterogeneity was observed for dietary EPA (I^2^ = 12% vs I^2^ = 36%) after omitting the only study^30^ for which variance-weighted least squares regression was used in the dose-response estimation. Subgroup dose-response analyses were not conducted for dietary n-3 PUFA intakes, but only for circulating n-3 PUFA concentrations, because the number of studies reporting dietary n-3 PUFAs in each subgroup was too small (not more than three) and the according exposure categories were very limited.

For circulating ALA exposure, even though one study^37^ contributed much of the weight in the continuous analysis, exclusion of this study did not change the pooled RR estimate (RR 1.00; 95% CI, 0.96–1.04; I^2^ = 27%). Sensitivity analysis showed that after omitting the study^30^ for which trend estimation was carried out using variance-weighted least squares regression as described above, the pooled RR of PCa was statistically significant for every 1% increase in blood DHA concentration (RR 1.02; 95% CI, 1.00–1.05), and the heterogeneity decreased from I^2^ = 26% to I^2^ = 16%. However, for blood EPA, exclusion of any individual study did not materially change the pooled estimates.

Subgroup analysis did not show any substantial change in the summary RR of trend estimation for circulating ALA exposure (eTable 7). However, for circulating DHA concentration, significant non-linear relationships with PCa risk were found among studies with longer follow-up (*P* = 0.04) or among studies conducted in Europe (*P* = 0.02) (Table 1). For blood concentration of EPA, five studies were conducted in the USA and all of these studies adjusted for age of participants; a significant non-linear association with PCa risk was observed in this subgroup (*P* < 0.01) (Table 2).

In studies with shorter follow-up, statistically significant pooled RRs of 1.03 (95% CI, 1.00–1.06; *P*_nonlinearity_ = 0.88) and 1.02 (95% CI, 1.00–1.04; *P*_nonlinearity_ = 0.40) were observed for every 1% increase in DHA concentration and every 0.5% increase in EPA concentration, respectively (Table 1 and Table 2). In addition, among studies that adjusted for BMI or alcohol consumption, both blood DHA and EPA showed significant positive associations with PCa risk (RR 1.03; 95% CI, 1.00–1.05 in BMI-adjusted subgroups and RR 1.03; 95 CI, 1.00–1.06 in alcohol consumption-adjusted subgroups for every 1% increase of DHA concentration; RR 1.02; 95% CI, 1.00–1.05 in both BMI-adjusted subgroups and alcohol consumption-adjusted subgroups for every 0.5% increase of EPA concentration) and no between-study heterogeneity was observed for any of these subgroups (I^2^ = 0%) (Table 1 and Table 2). For blood DPA, subgroup analysis indicated a significant inverse association with PCa risk (RR 0.97; 95% CI, 0.94–1.00, I^2^ = 43.5%) for every 0.2% increase in blood DPA concentration among studies with nested case-control designs. Moreover, the heterogeneity between these two subgroups (ie, nested case-control subgroups and case-cohort subgroups) was also significant (*P* < 0.01), which implied that study design may be a source of heterogeneity (eTable 8).

## DISCUSSION

To the best of our knowledge, this is the first dose-response meta-analysis to quantitatively evaluate the association between ALA and marine n-3 PUFA exposure and risk of PCa based on prospective studies. Even though all the food frequency questionnaires (FFQs) used in the included studies were validated by dietary records and were reported to have moderate to strong correlations between blood n-3 PUFA biomarkers and their dietary intakes assessed by FFQ, perfect correlations are unrealistic.^38^ There are also studies suggesting that biomarkers should be used to complement the FFQ rather than replace it, as biomarkers did not always perform better than the FFQ.^39^ Therefore, both blood biomarkers and dietary intakes of n-3 PUFA were separately examined for their association with PCa risk in the present study.

For ALA, previous studies did not report a conclusive association, and even meta-analyses have reported opposite conclusions (positively or negatively associated with PCa risk). Our present dose-response meta-analysis did not conservatively support a protective role of ALA, as the marginally significant negative association between PCa risk and per 0.5 g/day increment of dietary ALA was dominated by a single larger study and blood ALA concentration also showed no significant association with aggressive, non-aggressive, or total PCa risk. Higher dietary intakes of ALA did not result in higher physiologic levels of ALA in prostatic tissue^40^ and the complex metabolism of PUFAs, which may include beta-oxidation, storage in adipose tissues, incorporation into cell membranes, and bio-conversion to longer chain omega-3 PUFAs,^41^ may help explain the small heterogeneity of effects between dietary ALA and blood ALA. In addition, other potential reasons may help us explain the heterogeneity in the results of the present and previous studies. First, for the different original observational studies included, ALA can come from different dietary sources, such as vegetables and meat, which might lead to different types of confounding.^42^ Second, the quality of studies included in a meta-analysis plays a critical role in achieving reliable conclusions. As prospective studies are less prone to bias and could produce better evidence than retrospective studies, systematic reviews based on prospective studies may obtain inconsistent conclusions compared to systematic reviews that include retrospective studies. Even though blood ALA concentration showed no significant association with PCa risk, low ALA was reported as a factor for the preferred synthesis of AA from LA, instead of EPA from ALA, because competition exists between n-3 and n-6 for the respective synthesis of EPA and AA.^4^ Additionally, since the conversion rate from ALA to EPA and DPA in the body is very limited, dietary ALA is still the main source of circulating ALA.

For long chain n-3 PUFAs, which are present in fatty fish and fish oil, the median or average quantity of estimated exposure in extreme categories are listed in eTable 2 and eTable 3. People in Western countries did not always consume less long chain n-3 PUFAs than those in Asian countries. As reported by Hibbeln, dietary percentages of energy from long-chain n-3 fatty acids in Finland and Norway are comparable to those in Korea and much higher than those in China. Furthermore, people in Iceland have been reported to consume a much greater amount of long-chain fatty acids than people in Japan, who consumed the greatest amount of long chain n-3 PUFAs among Asian countries.^43^

Compared with the protective effects of long chain n-3 PUFAs reported in animal or in vitro studies, especially regarding EPA and DHA, results from epidemiological studies have been grossly inconsistent. At least two previous meta-analyses concluded that the blood levels of DHA and EPA were positively associated with PCa risk.^34^^,^^44^ Nevertheless, one of the studies was not based on prospective studies, and the other one did not include two recently published prospective studies with large sample sizes. In our study, the dose-response analysis using a random-effect model, which conservatively takes into account the heterogeneity between included studies, only found a borderline significant positive association between blood concentration of DHA and PCa risk. Moreover, this association was more significant after omitting a study that did not provide sufficient information regarding the distribution of cases used for trend estimation. On stratifying PCas by grade or stage, a borderline significant positive linear association was observed only for blood DHA with aggressive PCa risk. Nevertheless, both circulating DHA and EPA were positively associated with PCa risk in studies with shorter follow-up duration (less than 7.4 years), while circulating DHA and EPA were not associated with PCa risk in studies with longer follow-up periods (more than 7.4 years). Two reasons may help explain this heterogeneity: first, in order to avoid the high cost of fatty acid assays, most of the included nested case-control and case-cohort studies did not measure n-3 PUFA levels immediately after taking blood samples but instead stored the samples until the cases were diagnosed and matched controls were selected. As a result, longer follow-up means longer storage time, which may influence the precision of n-3 PUFA measurement; second, for assessment of dietary n-3 PUFA, different follow-up times may produce different levels of bias because the fatty acids database may not be regularly updated and other confounding constituents in the source of n-3 PUFA may influence the effects of n-3 PUFA in the long run.^45^

For dietary DHA exposure, we observed a significant non-linear association with PCa risk, which indicated a higher PCa risk with increasing intake of DHA. The discrepancies between the results of the present study and the protective effects reported in animal or in vitro studies may be explained by several reasons. First, the blood levels of marine n-3 PUFA in the population studied may be too low to produce observable protective effects, assuming that the protective effects of n-3 PUFA in preclinical studies of animal and cell culture models were true. Second, consumption of the main dietary source of DHA, fish, is very strongly related to socio-economic status and, more generally, to health-conscious behaviors. Undergoing screening for PCa is not only a very health-conscious behavior but is also known to increase the risk of being diagnosed with PCa (as is true of any screening method). Therefore, this bias may be involved in the positive association between DHA and PCa risk. Third, the background diet, which may contain n-6 PUFA or even potential carcinogenic substances, such as pesticides and heavy metals accumulated in fatty fish,^45^ may influence or even reverse the effects of marine n-3 PUFA consumption. Therefore, more prospective studies are warranted to investigate the impact of the n-3:n-6 ratio on cancers.

It is worth mentioning that even though DPA is present in smaller quantities than DHA and EPA in human blood, this fatty acid was reported to exhibit potent activities, such as reducing platelet aggregation, reducing age-related oxidation, inhibiting angiogenesis, and reducing inflammation, which may be involved in prostate carcinogenesis.^46^^–^^48^ In the present study, the pooled RR from 5 nested case-control studies indicated a significant inverse association between blood DPA concentration and total PCa risk. However, including another case-cohort study^34^ or stratifying the PCa by grade or stage eliminated this significant association. Therefore, more prospective studies examining the association between circulating or dietary DPA and PCa risk are needed.

One recent meta-analysis of prospective studies suggests that high BMI may be protective against localized PCa, while it was a risk factor for advanced PCa.^49^ Our subgroup analysis also indicates that BMI is an important confounding factor, because the positive association for both blood DHA and blood EPA with PCa risk is more evident in studies that adjusted for BMI. However, only two included studies assessed the association between blood DHA and PCa risk separately for localized and advanced PCa, which prevented us from performing further analysis on this interaction. Therefore, more studies assessing this confounding factor are needed, and the precise mechanisms for this interaction remain unclear. In addition, the adjustment for alcohol consumption also highlighted the positive association between blood DHA or blood EPA exposure and PCa risk, indicating that alcohol consumption may be involved in the incidence of PCa. However, several recent meta-analyses provided no consistent evidence on the association between alcohol drinking and PCa,^50^^–^^52^ and more studies are needed.

### Strengths and limitations

The present dose-response meta-analysis has several strengths. First, the large sample size and extensive information allowed us to quantitatively assess the dose-response association between ALA or other individual marine n-3 PUFA exposures and PCa risk, thus making it more powerful than any individual study. Second, the prospective nature of the included studies avoided the influence of recall and selection bias. Third, we systematically reviewed and assessed the summarized association between PCa with different types of individual n-3 PUFAs, including EPA, DHA, DPA, and ALA. Last, both blood level and dietary intakes of n-3 PUFAs were assessed for the association with PCa risk. These data give the most comprehensive view of the association between n-3 fatty acids and PCa risk based on current evidence.

The meta-analysis also has several limitations. First, when separately examining the findings for aggressive and non-aggressive PCa, available data on dietary intake of n-3 PUFAs is rather limited. Therefore, dose-response analysis for this aspect was not performed. In addition, subgroup analyses were not conducted for dietary n-3 PUFA exposure, because not more than three studies were eligible in each subgroup. Therefore, future prospective studies are needed for the detailed analysis of the association between dietary n-3 PUFA intake and risk of PCa. Second, the blood n-3 PUFA concentrations reflected exposure for only a short time period; therefore, this measurement may be subject to substantial random error. Last, possible language bias could occur because we excluded articles not in English.

### Conclusions

The present systematic review indicates that a significant negative association exists between ALA exposure and PCa risk, though the results were dominated by a single study. This finding does not support previous reports^20^ that high ALA intake or high ALA concentration is associated with increased PCa risk. However, both higher dietary DHA intakes and blood DHA concentrations were found to be associated with elevated risk of PCa, and each 1% increase in DHA concentration was associated with a 2% higher risk of PCa. When examining the findings stratified by the grade and stage of cancer, we observed that blood EPA concentrations and blood DHA concentrations were positively associated with aggressive PCa risk and nonaggressive PCa risk, respectively. Subgroup analyses indicated a 2% lower PCa risk per 1% increase in DPA concentration in studies with nested case-control study designs and suggested that study design, BMI, and alcohol consumption are important confounding factors. In general, different individual n-3 PUFAs may exhibit different or even opposite associations with PCa risk, and studies with larger sample sizes and longer follow-up times are needed to confirm the results.

## ONLINE ONLY MATERIALS

eTable 1. Electronic search strategies.

eTable 2. Characteristics of included studies for the association between dietary n-3 PUFAs and risk of prostate cancer.

eTable 3. Characteristics of included studies for the association between blood n-3 PUFAs and risk of prostate cancer.

eTable 4. Adjusted covariates for the included studies in the meta-analysis.

eTable 5. Case ascertainment of included studies in the meta-analysis.

eTable 6. Quality assessment of included studies on individual n-3 PUFAs and risk of prostate cancer.

eTable 7. Subgroup analyses per 0.1% increment of blood ALA concentration and risk of prostate cancer.

eTable 8. Subgroup analyses per 0.2% increment of blood DPA concentration and risk of prostate cancer.
