# Supplementary material for: Effect of Individual Omega-3 Fatty Acids on the Risk of Prostate Cancer: A Systematic Review and Dose-Response Meta-Analysis of Prospective Cohort Studies
Source: J Epidemiol. 2015 Apr 5;25(4):261–74. doi: 10.2188/jea.JE20140120 (PMC4375280; doi:10.2188/jea.JE20140120)
Supplement: eTable 1. [file je-25-261-s001.pdf]

eTable 1. Electronic search strategy

PubMed search

1. fat (Title/abstract)
2. fatty acid (Title/abstract)
3. docosahexaenoic acid (Title/abstract)
4. eicosapentaenoic acid (Title/abstract)
5. docosapentaenoic acid (Title/abstract)
6. alpha-linolenic acid (Title/abstract)
7. polyunsaturated fatty acid (Title/abstract)
8. omega-3 fatty acid (Title/abstract)
9. n-3 fatty acid (Title/abstract)
10. prostate cancer (Title/abstract)
11. prostate neoplasms (Title/abstract)
12. OR/1-9
13. OR/10-11
14. 12 AND 13
15. limit 14 to (English language and humans)
