# Supplementary material for: Effect of Individual Omega-3 Fatty Acids on the Risk of Prostate Cancer: A Systematic Review and Dose-Response Meta-Analysis of Prospective Cohort Studies
Source: J Epidemiol. 2015 Apr 5;25(4):261–74. doi: 10.2188/jea.JE20140120 (PMC4375280; doi:10.2188/jea.JE20140120)
Supplement: eTable 2. [file je-25-261-s002.pdf]

eTable 2. Characteristics of included studies for the association between dietary n-3 PUFA and risk of prostate cancer [1]

Study	Location	Patient Characteristics					Study type	Exposure measurement	n-3 PUFA type	Exposure range	Study Quality
		Follow-up (years)	Age (years) range or mean	Number of Participants	Number of Cases	Number of Controls					
Schuurman et al.[16] 1999	Netherlands	6.3	55-69	58,279	642		Case-cohort	Diet (g/day) 150-item FFQ	EPA	Highest: 0.10 g/day<br>Reference: 0	7
									DHA	Highest: 0.18 g/day<br>Reference: 0.01 g/day	
Wallström et al.[18] 2007	Sweden	11	45-73	10,564	817		prospective cohort	Diet (g/day) 168-item FFQ	ALA	Highest: 2.7 g/day<br>Reference: 1.4 g/day	9
									EPA	Highest: 0.44 g/day<br>Reference: 0.03 g/day	
									DHA	Highest: 0.86 g/day<br>Reference: 0.12 g/day	
									EPA+DHA	Highest: 1.3 g/day<br>Reference: 0.16 g/day	
									Total n-3	Highest: 3.8 g/day<br>Reference: 1.8 g/day	
Park et al.[17] 2007	USA	8.0	>45	82,483	4,404		Prospective cohort	Diet (g/1,000 kcal) >180-item FFQ	ALA	Highest: 2.14 g/day<br>Reference: 1.1 g/day	7
									EPA	Highest: 0.037 g/day<br>Reference: 0.008 g/day	
									DHA	Highest: 0.07 g/day<br>Reference: 0.01 g/day	

eTable 2. Characteristics of included studies for the association between dietary n-3 PUFA and risk of prostate cancer[1] (Continued)

									Total n-3	Highest: 2.26 g/day<br>Reference: 1.18 g/day	
Pelser et al.[31] 2013	USA	9	50-71	288,268	18,934		Prospective cohort	Diet (% energy) 124-item FFQ	ALA	Highest: 0.88%<br>Reference: 0.41%	7
									EPA	Highest: 0.036%<br>Reference: 0.003%	
									DHA	Highest: 0.0068%<br>Reference: 0.001%	
									DPA	Highest: 0.014%<br>Reference: 0	
									EPA+DHA	Highest: 0.103%<br>Reference: 0.013%	
									Total n-3	Highest: 0.95%<br>Reference: 0.45%	
Männistö[30] 2003	Finland	5-8	50-59	290,406	198	198	Nested case-control	Diet (g/day) 276-item FFQ	ALA	Highest: 2.3 g/day<br>Reference: 1.0 g/day	6
									DHA	Highest: 0.43 g/day<br>Reference: 0.11 g/day	
									EPA	Highest: 0.20 g/day<br>Reference: 0.05 g/day	
Giovannucci et al.[32] 2007	USA	16	40-75	51,529	3,544		Prospective cohort	Diet (g/day) 131-item FFQ	ALA	Highest: 1.43 g/day<br>Reference: 0.39 g/day	8

eTable 2. Characteristics of included studies for the association between dietary n-3 PUFA and risk of prostate cancer[1] (Continued)

Leitzmann et al.[33] 2004	USA	14	40-75	47,866	2,965		Prospective	Diet (% energy) 131-item FFQ	ALA	Highest: 0.62%<br>Reference: 0.19%	8
									EPA	Highest: 0.033%<br>Reference: 0.007%	
									DHA	Highest: 0.14%<br>Reference: 0.016%	
									EPA+DHA	Highest: 0.249%<br>Reference: 0.029%	
Koralek et al[13] 2011	USA	5.1	55-74	29,592	1,898		Prospective cohort	Diet (g/day) 137-item FFQ	ALA	Highest: 1.75 g/day<br>Reference: 1.38 g/day	8
Bassett et al.[27] 2013	Australia	8.9	27-80	17,045	464	1,717	Case-cohort	Diet (g/day) 121-item FFQ	ALA	Highest: 1.28 g/day<br>Reference: 0.79 g/day	6
									EPA	Highest: 0.19 g/day<br>Reference: 0.04 g/day	
									DHA	Highest: 0.33 g/day<br>Reference: 0.07 g/day	
									DPA	Highest: 0.07 g/day<br>Reference: 0.009 g/day	
									Total n-3	Highest: 1.75 g/day<br>Reference: 0.99 g/day	

[1] EPA, eicosapentaenoic acid; DHA, docosahexaenoic acid; DPA, docosapentaenoic acid; ALA, alpha-linolenic acid; FFQ, food frequency questionnaire.
