# Supplementary material for: Effect of Individual Omega-3 Fatty Acids on the Risk of Prostate Cancer: A Systematic Review and Dose-Response Meta-Analysis of Prospective Cohort Studies
Source: J Epidemiol. 2015 Apr 5;25(4):261–74. doi: 10.2188/jea.JE20140120 (PMC4375280; doi:10.2188/jea.JE20140120)
Supplement: eTable 3. [file je-25-261-s003.pdf]

eTable 3. Characteristics of included studies for the association between blood n-3 PUFA and risk of prostate cancer [1]

Study	Location	Follow-up (years)	Patient Characteristics: Age (years) range or mean	Number of Participants	Number of Cases	Number of Controls	Study type	Exposure measurement	n-3 PUFA type	Exposure range	Study Quality
Crowe et al.[37] 2008	Eight European Countries	4.2	cases: 60.4 controls: 60.1	137,001	962	1,061	Nested case-control	Plasma PL quintile	ALA	Highest: 1.50% Reference: 0.09%	7
									EPA	Highest: 1.65% Reference: 0.48%	
									DHA	Highest:4.97% Reference: 2.48%	
									DPA	Highest: 2.02% Reference: 0.71%	
Chavarro et al.[15] 2007	USA	13	40-84	14,916	476	476	Nested case-control	Whole blood quintile	ALA	Highest: 1.95% Reference: 0.24%	6
									EPA	Highest: 2.36% Reference: 1.28%	
									DHA	Highest: 3.37% Reference: 0.77%	
									DPA	Highest: 1.19% Reference: 0.77%	
									LC-n-3	Highest: 6.70% Reference: 3.66%	
Bassett [27] 2013	Australia	8.9	27-80	17,045	464	1,717	Case-cohort	Plasma PL quintile	ALA	Highest: 0.25% Reference: 0.09%	6

									EPA	Highest: 1.67%	

eTable 3. Characteristics of included studies for the association between blood n-3 PUFA and risk of prostate cancer[1] (Continued)

										Reference: 0.59%	
									DHA	Highest: 5.34%	
										Reference: 2.75%	
									DPA	Highest: 1.59%	
										Reference: 0.98%	
									total n-3	Highest: 8.38%	
										Reference: 5.10%	
Männistö et	Finland	5--8	50-69	290,406	198	198	Nested	Serum	ALA	Highest: 0.91%	6
al.[30]							case-control	quartile		Reference: 0.47%	
2003									DHA	Highest: 1.05%	
										Reference: 0.55%	
									EPA	Highest: 2.74%	
										Reference: 0.92%	
Harvei et al.[8]	Norway	11.6	50	NR	141	141	Nested	Serum	ALA	Highest: 0.21%	7
1997							case-control	quartile		Reference: 0.06%	
									EPA	Highest: 2.38%	
										Reference: 0.41%	
									DHA	Highest: 6.36%	
										Reference: 1.65%	
									DPA	Highest: 1.33%	
										Reference: 0.44%	
									total n-3	Highest: 9.07%	
										Reference: 5.23%	

eTable 3. Characteristics of included studies for the association between blood n-3 PUFA and risk of prostate cancer [1] (Continued)

Park et al.[36] 2009	USA	1.9	45-75	31,136	376	729	Nested case-control	Erythrocyte membrane quartile	ALA	Highest: 0.80% Reference: 0.17%	8
									EPA	Highest: 0.88% Reference: 0.21%	
									DHA	Highest: 8.73% Reference: 2.75%	
									DPA	Highest: 2.15% Reference: 0.63%	
									total n-3	Highest: 11.77% Reference: 4.00%	
Brasky et al[14] 2011	USA	7	55-84	18,882	1,658	1,803	Nested case-control	Serum PL quartile	ALA	Highest: 0.20% Reference: 0.06%	8
									EPA	Highest: 0.84% Reference: 0.22%	
									DHA	Highest: 3.58% Reference: 1.13%	
									EPA+DHA	Highest: 4.38% Reference: 1.39%	
Cheng et al.[35] 2013	USA	7	45-69	18,314	641	1,398	Nested case-control	Serum PL quartile	ALA	Highest: 0.12% Reference: 0.05%	8
									EPA	Highest: 0.84% Reference: 0.22%	
									DHA	Highest: 3.43%	

Reference: 1.06%

eTable 3. Characteristics of included studies for the association between blood n-3 PUFA and risk of prostate cancer 1 (Continued)

									DPA	Highest:0.95%	
										Reference: 0.36%	
									total n-3	Highest: 4.80%	
										Reference: 3.53%	
Brasky et al.[34]	USA	6	>50	35,533	834	1,393	case-cohort	Plasma PL	ALA	Highest: 0.19%	8
2013								quartile		Reference: 0.05%	
									EPA	Highest: 0.94%	
										Reference: 0.22%	
									DHA	Highest: 3.96%	
										Reference: 1.17%	
									DPA	Highest: 1.05%	
										Reference: 0.38%	
									LC-n-3	Highest: 5.76%<br>Reference: 1.84	

[1] EPA, eicosapentaenoic acid; DHA, docosahexaenoic acid; DPA, docosapentaenoic acid; ALA, alpha-linolenic acid; PL, phospholipid.
