# Supplementary material for: Effect of Individual Omega-3 Fatty Acids on the Risk of Prostate Cancer: A Systematic Review and Dose-Response Meta-Analysis of Prospective Cohort Studies
Source: J Epidemiol. 2015 Apr 5;25(4):261–74. doi: 10.2188/jea.JE20140120 (PMC4375280; doi:10.2188/jea.JE20140120)
Supplement: eTable 4. [file je-25-261-s004.pdf]

eTable 4. Adjusted covariates for the included studies in the meta-analysis

Study source	Adjusted covariates in each included study
Crowe 2008, Europe [37]	Body mass index, smoking, alcohol intake, education, marital status, and physical activity
Harvei 1997, Norway [8]	No adjustment
Männistö 2003, Finland [30]	No adjustment
Park 2009, USA [36]	Age at blood draw, fasting hours prior to blood draw, family history of prostate cancer, BMI, and education
Chavarro 2007, USA [15]	Age, smoking status at baseline, and length of follow-up
Brasky 2011, USA [14]	Age, race, family history of prostate cancer, diabetes, body mass index, alcohol, and treatment arm
Bassett 2013, Australia [27]	Country of birth, education, alcohol intake, physical activity, total energy intake from food and family history of cancer and smoking status
Cheng 2013, USA [35]	Age at enrollment, race, family history of prostate cancer in first-degree relatives, alcohol consumption, smoking status, smoking pack-years, and BMI
Brasky 2013, USA [34]	Age, race, education, history of diabetes, family history of prostate cancer, and SELECT intervention arm
Schuurman 1999,[16]	Age, family history of prostate carcinoma, socioeconomic status, total energy intake, and total energy-adjusted fat intake
Koralek 2006, USA [13]	Age, BMI, family history of prostate cancer, history of diabetes, smoking history, intake of total energy, lycopene, supplemental vitamin E, aspirin use, physical activity, number of screens, study center, and race
Giovannucci 2007, USA [32]	Age, time period, BMI at 21 years, height, cigarette pack-years in previous 10 years, vigorous physical activity level, family history of prostate cancer, history of diabetes mellitus, race, and intakes of total calories, processed meat, fish, ALA, tomato sauce, and vitamin E supplements
Leitzmann 2004, USA [33]	Current age, time period, major ancestry, family history of prostate cancer, BMI at age 21y, height, history of type 2 diabetes, history of vasectomy, cigarette smoking in the previous decade, vigorous physical activity, intake of total energy, percentage of energy from trans unsaturated fat intake, and intake of calcium, supplemental vitamin E, and lycopene
Park 2007, USA [17]	Time on study, ethnicity, family history of prostate cancer, education, BMI, smoking status and energy intake
Wallström 2007, Sweden [18]	Age, diabetes, waist circumference, height, living alone/with partner/with other (categorical), educational level (ordinal), alcohol habits (categorical), BMI(categorical), smoking history (ordinal), birth country (Sweden/other, categorical), total calcium intake, consumption of fruits, vegetables, red meat, energy
Pelser 2013, USA [31]	Age at entry, race, family history of prostate cancer, education, marital status, PSA testing in the past 3 years, physical activity, smoking, self-reported diabetes, BMI at baseline, calories, alcohol, and intake of tomatoes
