# Supplementary material for: Effect of Individual Omega-3 Fatty Acids on the Risk of Prostate Cancer: A Systematic Review and Dose-Response Meta-Analysis of Prospective Cohort Studies
Source: J Epidemiol. 2015 Apr 5;25(4):261–74. doi: 10.2188/jea.JE20140120 (PMC4375280; doi:10.2188/jea.JE20140120)
Supplement: eTable 5. [file je-25-261-s005.pdf]

## eTable 5. Case ascertainment of included studies in the meta-analysis

| Study source | Case ascertainment |
| --- | --- |
| Crowe 2008, Europe [37] | Dutch National Cancer Registry and the Regional Cancer Registries Amsterdam, East, and Limburg provided data on cancer incidence. |
| Harvei 1997, Norway [8] | Identified by matching the serum bank files with the Cancer Registry, using the unique 11-digit personal identification number that is allocated to every citizen of Norway. |
| Männistö 2003, Finland [30] | Ascertained using the Finnish Cancer Registry and the Register of Causes of Death. |
| Park 2009, USA [36] | Ascertained through the tumor registries of the Surveillance, Epidemiology and End Results (SEER) Program of the National Cancer Institute covering Hawaii and California. |
| Chavarro 2007, USA [15] | By requesting hospital records and pathology reports for review by study physicians from the End Points Committee. |
| Brasky 2011, USA [14] | By prostate biopsy. |
| Bassett 2013, Australia [27] | Identified by notifications of first diagnoses of prostate cancer to the Victorian Cancer Registry or the Australian Cancer Database. |
| Cheng 2013, USA [35] | By review of medical records and pathology reports in Western Washington Cancer Surveillance System, a part of the Surveillance, Epidemiology, and End Results registries. |
| Brasky 2013, USA [34] | By obtaining pathology reports and, when possible, tissue. Almost all cases included were reviewed centrally for pathological confirmation and grading using the Gleason system. |
| Schuurman 1999,[16] | Detected by computerized record linkage with all nine cancer registries in The Netherlands and with the Dutch national data base of pathology reports. |
| Koralek 2006, USA [13] | Autopsy data, supporting medical/pathologic records were used to confirm the diagnosis and stage and grade information. |
| Giovannucci 2007, USA [32] | Ascertained by relevant hospital records and pathology reports. |
| Leitzmann 2004, USA [33] | Using medical records and pathology reports to confirm the diagnosis and obtain further details. |
| Park 2007, USA [17] | Identified by linkage of the cohort to 3 population-based cancer registries in Hawaii and California that are all members of the National Cancer Institute's Surveillance. |
| Wallström 2007, Sweden [18] | Ascertained by record linkage with the National Cancer Register (Sweden). |
| Pelser 2013, USA [31] | Ascertained by linkage to 8 state cancer registries and 3 additional state cancer registries (Arizona, Nevada, and Texas). |
