# Supplementary material for: Effect of Individual Omega-3 Fatty Acids on the Risk of Prostate Cancer: A Systematic Review and Dose-Response Meta-Analysis of Prospective Cohort Studies
Source: J Epidemiol. 2015 Apr 5;25(4):261–74. doi: 10.2188/jea.JE20140120 (PMC4375280; doi:10.2188/jea.JE20140120)
Supplement: eTable 6. [file je-25-261-s006.pdf]

eTable 6. Quality assessment of included studies on individual n-3 polyunsaturated fatty acids and risk of prostate cancer[1]

Study	Selection	Comparability	Outcome/Exposure	Overall quality
Harvei et al.[8], 1997, Norway	4	0	3	7
Schuurman et al.[16], 1999, Netherlands	4	1	2	7
Männistö et al.[30], 2003, Finland	3	0	3	6
Leitzmann et al.[33], 2004, USA	3	2	3	8
Koralek et al.[13], 2006, USA	4	2	2	8
Chavarro et al.[15], 2007, USA	3	1	2	6
Park et al.[17], 2007, USA	4	1	2	7
Wallström et al.[18], 2007, Sweden	4	2	3	9
Givannucci et al.[32], 2007, USA	3	2	3	8
Crowe et al.[37], 2008, European countries	4	1	2	7
Park et al.[36], 2009, USA	4	2	2	8
Brasky et al.[14], 2011, USA	4	2	1	7
Brasky et al.[34], 2013, USA	4	1	2	7
Cheng et al.[35], 2013, USA	4	2	2	8
Bassett et al.[27], 2013, Australia	3	0	3	6
Pelser et al.[31], 2013, USA	3	2	2	7

[1]The study quality was assessed according to the Newcastle Ottawa Quality assessment scale for cohort studies and case-control studies. The scale awards a maximum of 9 points to each study: 4 for selection, 2 for comparability, and 3 for assessment of outcomes (for cohort study) or exposures (for case-control study). Comparability was assessed based on the adjustment of age and BMI
