# Supplementary material for: Effect of Individual Omega-3 Fatty Acids on the Risk of Prostate Cancer: A Systematic Review and Dose-Response Meta-Analysis of Prospective Cohort Studies
Source: J Epidemiol. 2015 Apr 5;25(4):261–74. doi: 10.2188/jea.JE20140120 (PMC4375280; doi:10.2188/jea.JE20140120)
Supplement: eTable 7. [file je-25-261-s007.pdf]

eTable 7. Subgroup analyses for per 0.1% increment[a] of blood ALA[b] concentration and risk of prostate cancer

Subgroup factor		Number of studies	Relative risk (95% CI)	Heterogeneity within sub-group		Heterogeneity between sub-groups	Nonlinearity
				I^2	Degree	p value	p value
**Overall analysis**		9	1.00 (0.98, 1.03)	18%	-	-	0.70
**Study design**							
	Nested case-control	7	1.01（0.98, 1.04)	24%	Low	0.21	0.53
	Case-cohort	2	-	-		-	-
**Grade or stage of cancer**							
	Aggressive	5	0.98 (0.87, 1.11)	42%	Moderate	-	0.44
	Non-aggressive	4					<0.01
**Follow-up duration**							
	≤7 years	6	1.00 (0.99, 1.02)	18%	Low	0.22	0.98
	>7 years	3	1.06 (0.94, 1.20)	58%	Moderate		0.62
**Regions**							
	USA	5	1.00 (0.98, 1.03)	58%	Moderate	0.56	0.57
	European countries	3	1.02 (0.97, 1.06)	8%	Low		
	Australia	1	-	-	-	-	-
**Covariate adjustment**							
	Adjusted for age	5	1.00 (0.95, 1.04)	8%	Low	0.58	0.57
	Not adjusted for age	4	1.01 (0.97, 1.05)	41%	Moderate		0.68
	Adjusted for BMI	4	1.01 (0.99, 1.03)	0%	Low	0.80	0.69
	Not adjusted for BMI	5	1.00 (0.94, 1.07)	53%	Moderate		0.94
	Adjusted for alcohol consumption	4	1.01 (0.99, 1.03)	0%	Low	0.90	0.79
	Not adjusted for alcohol consumption	5	1.01 (0.95, 1.06)	52%	Moderate		0.38

Adjusted for smoking status	4	1.01 (0.99, 1.03)	0%	Low	0.30	0.49
Not adjusted for smoking status	5	0.99 (0.94, 1.04)	44%	Moderate		0.39
Adjusted for family history of prostate cancer	4	1.02 (0.98, 1.06)	47%	Moderate	0.11	0.55
Not adjusted for family history of prostate cancer	5	- -	-	-		0.02
**Risk expression**						
Hazard/rate ratio	2	-	-		-	-
Relative risk	2	-	-		-	-
Odds ratio	5	1.00 (0.95, 1.06)	39%	Moderate	0.57	0.22

BMI, body mass index; CI, confidence interval; PCa, prostate cancer.

[a] The range of blood ALA concentration in the included studies is 0.06% to 1.49%

[b] ALA: alpha-linolenic acid
