# Supplementary material for: Effect of Individual Omega-3 Fatty Acids on the Risk of Prostate Cancer: A Systematic Review and Dose-Response Meta-Analysis of Prospective Cohort Studies
Source: J Epidemiol. 2015 Apr 5;25(4):261–74. doi: 10.2188/jea.JE20140120 (PMC4375280; doi:10.2188/jea.JE20140120)
Supplement: eTable 8. [file je-25-261-s008.pdf]

eTable 8. Subgroup analyses per 0.2% increment[a] of blood DPA[b] concentration and risk of prostate cancer

Subgroup factor	Number of studies	Relative risk (95% CI)	Heterogeneity within sub-group		Heterogeneity between sub-groups	Nonlinearity
			I^2	Degree	p value	p value
**Overall analysis**	7	0.99 (0.96, 1.02)			-	
**Study design**						
Nested case-control	5	0.97 (0.94, 1.00)	44%	Moderate	<0.01	0.28
Case-cohort	2	-	-		-	-
**Follow-up duration**						
≤7 years	4	1.00 (0.96, 1.04)	71%	Moderate	0.11	0.21
>7 years	3	0.96 (0.91, 1.01)	62%	Moderate		0.64
**Regions**						
USA	4	0.99 (0.94, 1.04)	78%	High	0.78	0.06
European countries	2	-	-	-	-	-
Australia	1	-	-	-	-	-
**Covariate adjustment**						
Adjusted for age	4	0.99 (0.94, 1.04)	78%	High	0.78	0.06
Not adjusted for age	3	0.99 (0.95, 1.03)	57%	Moderate		0.91
Adjusted for BMI	3	0.99 (0.96, 1.01)	18%	Low	0.50	0.31
Not adjusted for BMI	4	0.98 (0.92, 1.05)	80%	High		0.33
Adjusted for alcohol consumption	3	1.00 (0.97, 1.02)	0%	Low	0.52	0.68
Not adjusted for alcohol consumption	4	0.97 (0.91, 1.04)	82%	High		0.08
Adjusted for smoking status	4	0.99 (0.96, 1.01)	23%	Low	0.72	0.79
Not adjusted for smoking status	3	0.98 (0.90, 1.07)	86%	High		0.15

Adjusted for family history of prostate cancer	3	0.96 (0.90, 1.02)	70%	Moderate	0.29	0.91
Not adjusted for family history of prostate cancer	4	1.00 (0.96, 1.04)	71%	Moderate		0.08
**Risk expression**						
Hazard/rate ratio	2	-	-		-	-
Relative risk	2	-	-		-	-
Odds ratio	3	0.96 (0.93, 0.99)	7%	Low	0.02	0.06

BMI, body mass index; CI, confidence interval; PCa, prostate cancer.

[a] The range of blood ALA concentration in the included studies is 0.06% to 1.49%

[b] DPA: docosapentaenoic acid
